# Supplementary material for: Night-Time Exposure to Road, Railway, Aircraft, and Recreational Noise Is Associated with Hypnotic Psychotropic Drug Dispensing for Chronic Insomnia in the Paris Metropolitan Area
Source: Int J Environ Res Public Health. 2025 Oct 30;22(11):1647. doi: 10.3390/ijerph22111647 (PMC12652590; doi:10.3390/ijerph22111647)
Supplement: Supplementary file 1 [file ijerph-22-01647-s001.zip › Table S1.pdf]

Table S1. Hypnotic psychotropic drugs selected and dispensed in the study area (2017-2019), grouped by their Anatomical Therapeutic Chemical (ATC) code.

ATC classification	Drugs (N)	Dispensing N (%)
**Antihistamines for systemic use (R06)**	**6**	**670 555 (2,8%)**
R06AD01 – Alimemazine*	4	667 690
R06AD02 - Promethazine	2	2 865
**Psychoanaleptics (N06)**	**87**	**1 500 254 (6,3%)**
N06AA06 - Trimipramine	4	14 497
N06AA09 - Amitriptyline	6	482 464
N06AA12 - Doxepin	5	5 128
N06AX03 - Mianserin	40	556 724
N06AX11 - Mirtazapine	23	394 517
N06AX22 - Agomelatine	9	46 924
**Psycholeptics (N05) and related drugs**	**276**	**21 007 553 (88,3%)**
N05BA01 – Diazepam	14	761 943
N05BA04 – Oxazepam	4	1 143 642
N05BA05 – Clorazepate	8	262 141
N05BA06 – Lorazepam*	11	1 374 334
N05BA08 – Bromazepam	29	3 138 101
N05BA09 – Clobazam	3	359 944
N05BA11 – Prazepam	7	1 075 354
N05BA12 – Alprazolam	61	3 802 808
N05BA18 – Ethyl loflazepate	2	67 157
N05BA21 – Clotiazepam	2	134 380
N05BB01 – Hydroxyzine*	24	1 892 114
N05CD02 – Nitrazepam*	2	26 078
N05CD04 – Estazolam*	1	38 694
N05CD06 – Lormetazepam*	6	824 832
N05CD11 – Loprazolam*	1	225 627
N05CF01 – Zopiclone*	53	3 502 972
N05CF02 – Zolpidem*	43	2 376 905
N05CF04 – Eszopiclone*	3	0
N05CM09 – Valerian*	2	527
**Other drugs**	**12**	**623 014 (2,6%)**
V03AX - Other therapeutic products [1]*	9	620 474
Z - No ATC code assigned[2]*	3	2 540
**TOTAL**	**381**	**23 801 376**

[1]Hawthorn, Valerian, Passionflower; [2]Combination of valerian, passionflower, hawthorn, and black horehound; *Drugs with a marketing authorization granted by the French Medicines Agency specifically for sleep disorders or insomnia.
