# Supplementary material for: Night-Time Exposure to Road, Railway, Aircraft, and Recreational Noise Is Associated with Hypnotic Psychotropic Drug Dispensing for Chronic Insomnia in the Paris Metropolitan Area
Source: Int J Environ Res Public Health. 2025 Oct 30;22(11):1647. doi: 10.3390/ijerph22111647 (PMC12652590; doi:10.3390/ijerph22111647)
Supplement: Supplementary file 1 [file ijerph-22-01647-s001.zip › Table S2.pdf]

Table S1. Change (with 95% CIs) in prevalence of patients (/1,000 inhabitants) reimbursed for hypnotic psychotropic drugs per 5 dB(A) increase in night-time noise level (AEI L_n) from all noise sources combined (road, rail, aircraft and recreational).

	Women	Men	
	Estimate (β) [95% CI]	*Estimate (β) [95% CI]*	*p-value*
**Age groups**			
18-34	0.79 [0.79 – 0.80]	-2.86 [-2.90 – -2.82]	< 0.001
35-49	2.47 [2.41 – 2.52]	-1.33 [-1.40 – -1.25]	< 0.001
50-64	5.43 [5.38 – 5.48]	3.47 [3.40 – 3.55]	< 0.001
65-79	7.79 [7.74 – 7.84]	7.29 [7.21 – 7.36]	< 0.001
**Deprivation index (quintile)**			
1	2.11 [2.09 – 2.12]	0.09 [0.02 – 0.15]	< 0.001
2	2.22 [2.15 – 2.28]	-0.43 [-0.56 – -0.31]	< 0.001
3	2.39 [2.33 – 2.46]	-0.08 [-0.21 – 0.05]	< 0.001
4	2.58 [2.52 – 2.65]	0.23 [0.10 – 0.36]	< 0.001
5	2.74 [2.67 – 2.81]	0.41 [0.29 – 0.54]	< 0.001

Notes: Models included a three-way interaction — (1) sex × age × combined exposure to all noise sources, and (2) sex × deprivation index quintile × combined exposure to all noise sources, in addition to the following confounders: proportion of the population reporting a primary care physician, and log-transformed population density. Quintile 1 = least disadvantaged IRIS; quintile 5 = most disadvantaged IRIS. *Abbreviations*: CI, confidence intervals; AEI, average energetic index.
