# Supplementary figures and images for: Night-Time Exposure to Road, Railway, Aircraft, and Recreational Noise Is Associated with Hypnotic Psychotropic Drug Dispensing for Chronic Insomnia in the Paris Metropolitan Area
Source: Int J Environ Res Public Health. 2025 Oct 30;22(11):1647. doi: 10.3390/ijerph22111647 (PMC12652590; doi:10.3390/ijerph22111647)

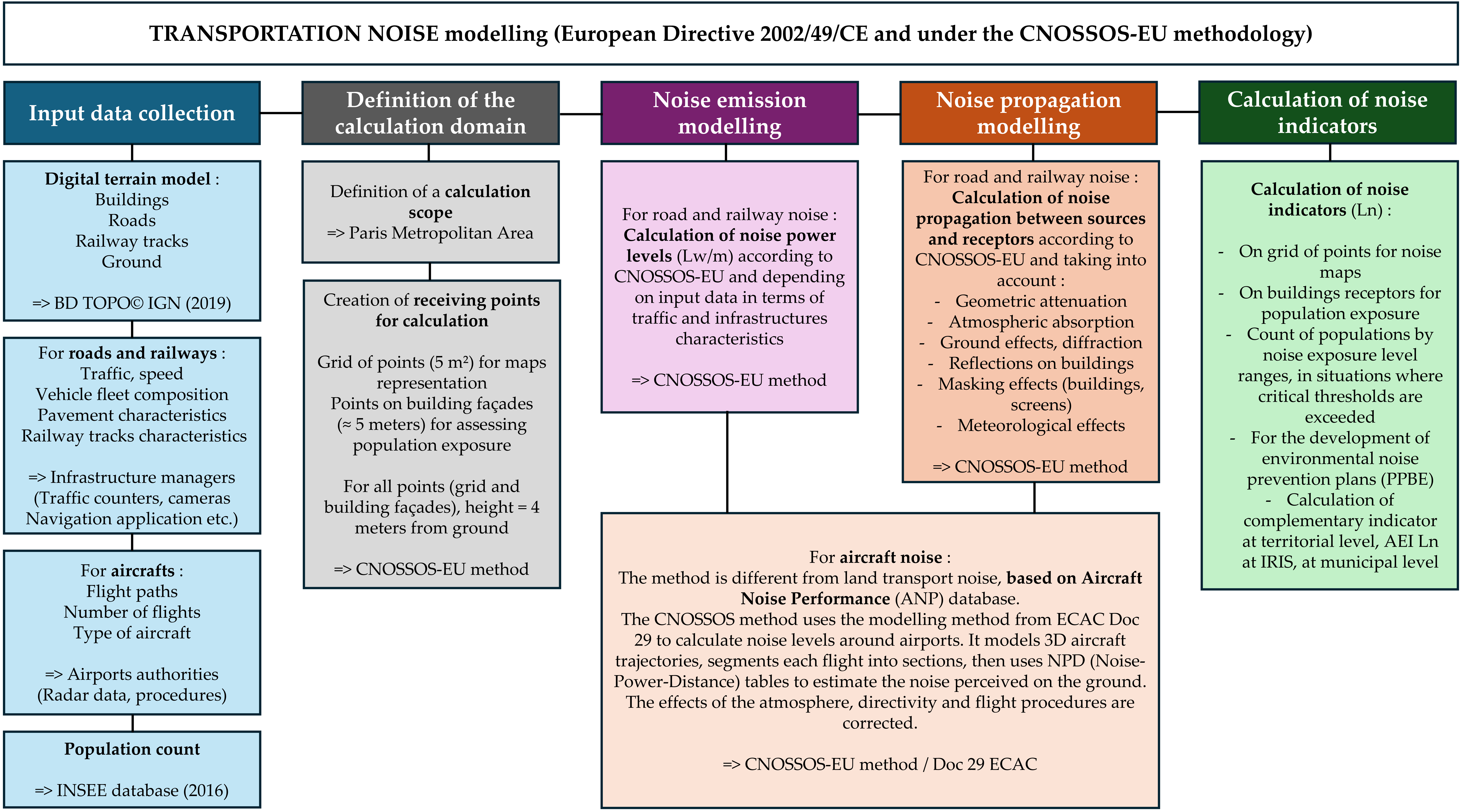

Supplement: Supplementary file 1 [file ijerph-22-01647-s001.zip › Figure S1.png]

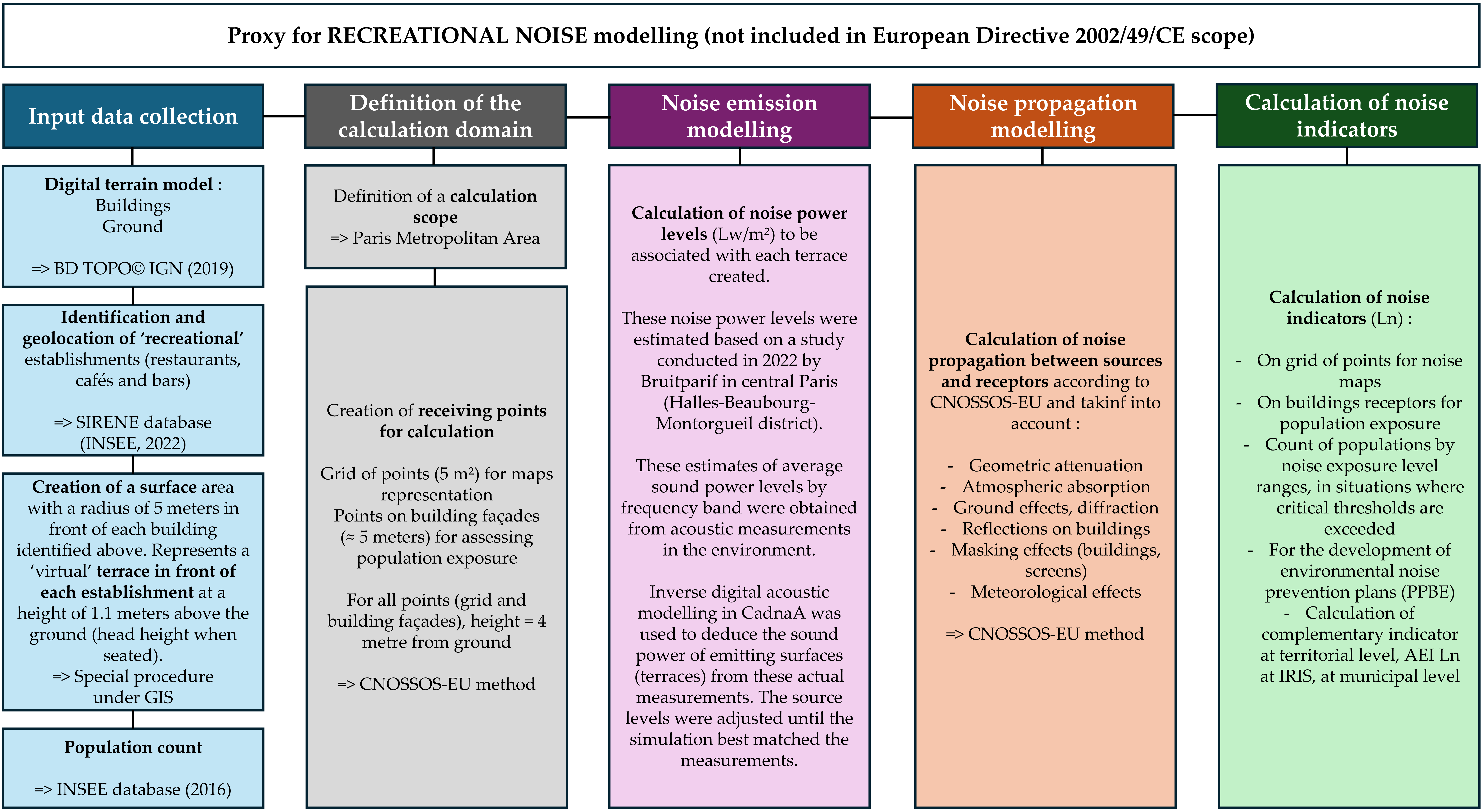

Supplement: Supplementary file 1 [file ijerph-22-01647-s001.zip › Figure S2.png]

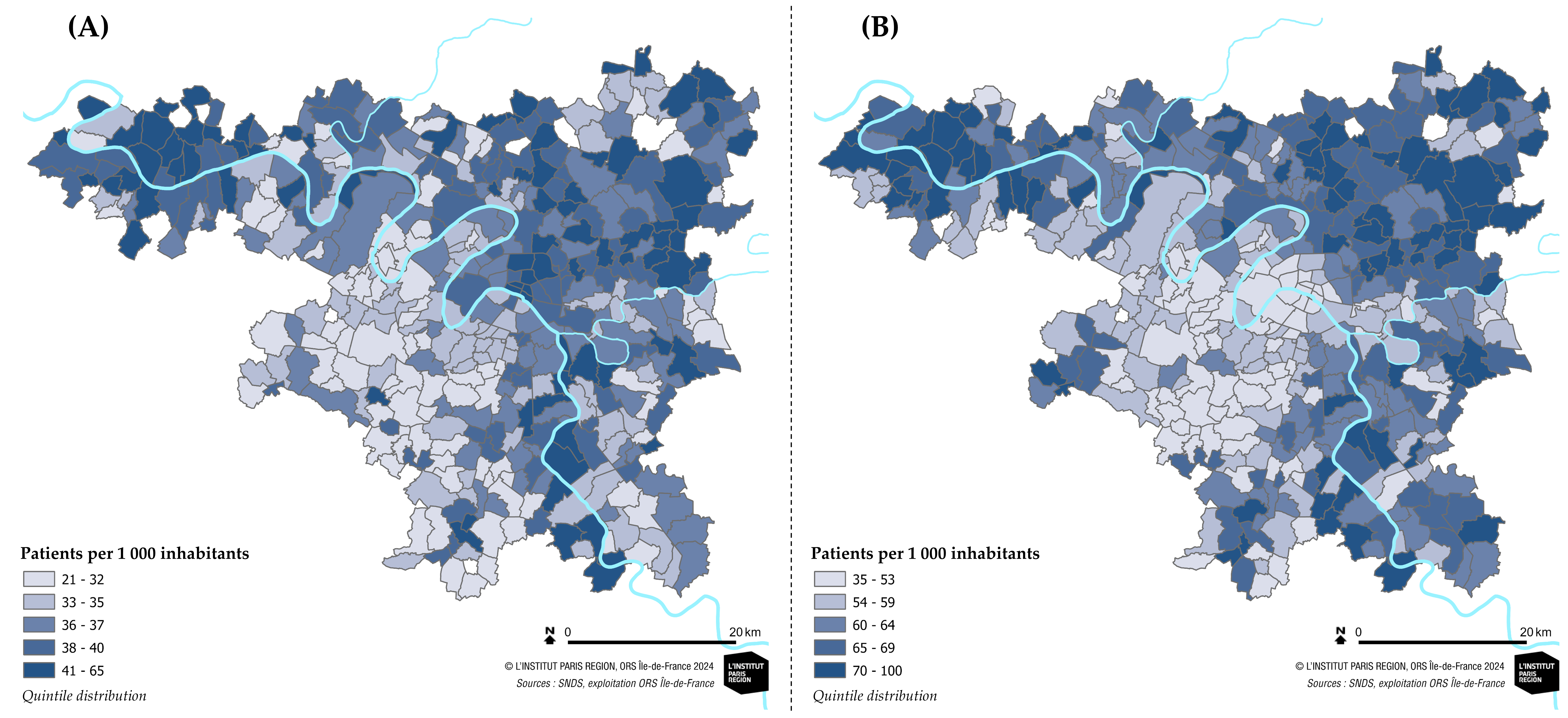

Supplement: Supplementary file 1 [file ijerph-22-01647-s001.zip › Figure S3.png]
